# Flos Puerariae Extract Prevents Myocardial Apoptosis via Attenuation Oxidative Stress in Streptozotocin-Induced Diabetic Mice

**DOI:** 10.1371/journal.pone.0098044

**Published:** 2014-05-27

**Authors:** Wei Yu, Wenliang Zha, Shuang Guo, Hongke Cheng, Jiliang Wu, Chao Liu

**Affiliations:** Hubei Province Key Laboratory on Cardiovascular, Cerebrovascular, and Metabolic Disorders, Hubei University of Science and Technology, Xianning, China; University of Western Ontario, Canada

## Abstract

**Background:**

Diabetic cardiomyopathy (DCM) suggests a direct cellular insult to myocardium. Apoptosis is considered as one of the hallmarks of DCM. Oxidative stress plays a key role in the pathogenesis of DCM. In this study, we explored the prevention of myocardial apoptosis by crude extract from *Flos Puerariae* (FPE) in experimental diabetic mice.

**Methods:**

Experimental diabetic model was induced by intraperitoneally injection of streptozotocin (STZ, 50 mg/kg/day) for five consecutive days in C57BL/6J mice. FPE (100, 200 mg/kg) was orally administrated once a day for ten weeks. Cardiac structure changes, apoptosis, superoxide production, NADPH oxidase subunits expression (gp91^phox^, p47^phox^, and p67^phox^), and related regulatory factors were assessed in the heart of mice.

**Results:**

Diabetic mice were characterized by high blood glucose (≥11.1 mmol/L) and reduced body weight. In the end of the experiment, aberrant myofilament structure, as well as TUNEL positive cardiac cells coupled with increased Bax/Bcl-2 ratio and Caspase-3 expression was found in diabetic mice. Moreover, ROS formation, the ratio of NADP^+^/NADPH and NADPH oxidase subunits expression of gp91^phox^ and p47^phox^, lipid peroxidation level was significantly increased, while antioxidant enzyme SOD and GSH-Px activity were reduced in the myocardial tissue of diabetic mice. In contrast, treatment with FPE resulted in a normalized glucose and weight profile. FPE administration also preserved myocardial structure and reduced apoptotic cardiac cell death in diabetic mice. The elevated markers of oxidative stress were significantly reversed by FPE supplementation. Further, FPE treatment markedly inhibited the increased Bax/Bcl-2 ratio and Caspase-3 expression, as well as suppressed JNK and P38 MAPK activation in the heart of diabetic mice.

**Conclusions:**

Our data demonstrate for the first time that FPE may have therapeutic potential for STZ-induced diabetic cardiomyopathy through preventing myocardial apoptosis via attenuation oxidative stress. And this effect is probably mediated by JNK and P38 MAPK signaling pathway.

## Introduction

Accumulating data from epidemiologic, experimental, and clinical studies have shown that diabetes results in structural and functional cardiac changes. And individuals with diabetes have a cardiac mortality equivalent to that in nondiabetic patients with confirmed heart disease. Diabetic cardiomyopathy (DCM) was initially put forward by Rubler in 1972 [1]. It is defined as ventricular dysfunction occurring independently of a recognized cause such as CAD or hypertension [2]. The concept of DCM suggests a direct cellular insult to the myocardium. It may affect the myocardium secondary to diabetes causing a synergistic adverse effect, as seen with a combination of diabetes and vascular complications. 

Apoptotic cell death is increased in the diabetic heart of patients and animal models [3], [4]. In diabetic cardiomyopathy (DCM), cell apoptosis is generally accepted as the etiological factor and closely related to cardiac ROS generation. And treatment with antioxidants protected cardiomyocytes from apoptosis in high glucose conditions [5], [6]. Reactive oxygen species (ROS) derived from hyperglycemia is considered to be the most important contributor in the development and the progression of DCM [7]. Oxidative stress occurs when ROS production overweighs their degradation by antioxidant defenses, and leads to numerous deleterious effects via cellular damage by oxidation and disruption vascular hemostasis, as well as by modulation of detrimental intracellular signaling pathways on the cardiovascular system [3], [8].Given the key role of excessive oxidative stress on the pathogenesis of diabetic heart, there is growing interest in antioxidants used as a compensatory therapeutic approach.

*Flos Puerariae*, a well-known Chinese medicine compound widely cultivated in East Asian, is the dry bud of Pueraria lobata that a plant in the genus Pueraria of the pea family Fabaceae, subfamily Faboideae. Different active constituents extracted from *Flos Puerariae* have been proved to possess a lot of activities such as antioxidant, hypoglycemic, hypolipidemic, antidiabetic, et al. Recently we have established an optimum condition for total flavonoids extracted from *Flos Pueraria* in our laboratory. And the yield of total flavonoids in the final extracts has been proved up to 17.5%. There are at least five isoflavones has been separated and identified as irisolidone, genistein, daidzein, kakkalide and puerarin, respectively. Previous study from our lab has demonstrated this crude extract from *Flos Puerariae* ameliorated retinopathy by inhibiting apoptosis in diabetic mice [9]. And it aroused our interests to explore if FPE play a role on myocardial apoptosis during the development and progression of DCM.

In the present study, we have set up an experimental diabetic model by multiple injection of streptozotocin (STZ, 50 mg/kg/day) for five consecutive days in C57BL/6J mice. The aim of this study is to investigate whether FPE has a protective effect against myocardial apoptosis and the underlying mechanisms in experimental diabetic mice.

## Materials and Methods

### Preparation of Flos Puerariae Extract (FPE)

The crude extract of *Flos Puerariae* was prepared at the Phytochemistry Laboratory, Department of Materia Medica, Hubei University of Science and Technology. The dried aerial part of *Flos Puerariae* was mixed with 50% (V/V) methanol solution with the solid-to-liquid ratio at 1∶30. The mixture was extracted with ultrasound for 2 h at 70°C. The extracted products was then purified sequentially by petroleum ether, ethanol and chloroform-butyl alcohol, and eluted gradually with mixed mobile phase of methanol-chloroform solution in the silica gel column system. In the end, the isolated ingredients were further analyzed by color reaction, ultraviolet spectrophotometry, high performance liquid chromatography, infrared spectrum and mass spectrum. Total flavonoids in the final extract were proved up to 17.5%. And five primary isoflavones were identified as irisolidone, genistein, daidzein, kakkalide and puerarin [10].

### Experimental animals

Sixty male C57BL/6J mice (22±2 g) were purchased from the Laboratory Animal Center of Wuhan University. Mice were housed with a 12 h light/dark cycle, at temperature of 23∼25°C and humidity of 55∼60%. All animals were treated in accordance with the *Guide for the Care and Use of Laboratory Animals* published by the US National Institutes of Health(NIH Publication No. 85–23, revised 1996). All experiments were approved by IACUC (Institutional Animal Care and Use Committee of Huazhong University of Science and Technology) under permit number 2012-S300.

### Induction of experimental diabetic model

Before experiments, all mice were adaptive feeding for one week. All the animals were provided with food and water *ad libitum*. Diabetic mice model was set up by intraperitoneally injection with STZ (Sigma, USA) according to previous description with little modification [11], [12]. Briefly, mice were intraperitoneally injected with STZ in sodium citrate buffer (pH 4.5) for 5 consecutive days (50 mg/kg/day). Control mice were administered an equivalent volume of citrate buffer. Blood glucose levels were measured after STZ injection using hand-held glucometer (Changsha Sinocare Inc. China) by tail vein blood sampling. Those mice with blood glucose levels ≥11.1 mmol/L were included for the next experiment. In the fourth week after STZ injection, experimental mice were randomly divided into 4 groups: Control group, DM group, and FPE groups (HFPE: high dose, 200 mg/kg; LFPE: low dose, 100 mg/kg). FPE were orally administrated in the drinking water for 10 weeks in FPE groups. Control and DM group were treated with equal-volume of saline.

### Sample collection

After experiment, all animals were sacrificed. Whole blood was collected and the serum was separated. The hearts were excised. Atria and right ventricle were removed. Tissues were stored in −80°C for biochemical analysis, or placed in 4% paraformaldehyde for paraffin embedding, or fixed in 2.5% glutaraldehyde fixative for electronic speculum detection.

### H&E staining

Paraffin-embedded tissues were sectioned (4 µm thickness) for histological analysis. Hematoxylin-eosin (H&E) stained sections were photographed under light microscopy at×400 magnification.

### Electron microscopy observation

After fixation in 2.5% glutaraldehyde fixative followed by post fixation in 1% osmium tetroxide solution, small pieces of heart were rinsed and dehydrated in ascending alcohol series. Samples were then embedded in epon and cut on an ultramicrotome, and finally double-stained with uranyl acetate and lead citrate. The sections were observed by transmission electron microscope (FEI Tecnai G^2^12).

### Terminal deoxynucleotidyl transferase-mediated dUTP nick end-labelling (TUNEL) assay

The paraffin samples were removed from the sections with xylene, rehydrated in graded alcohol series, and then placed in 3% hydrogen peroxide in methanol for 10 min at room temperature. Sections were then incubated with 20 mg·mL^−1^ proteinase K for 15 min. The sections were washed several times in PBS and then incubated with equilibration buffer for 15 s and TdT-enzyme at 37°C for 1 h. Antibody blocking then proceeded for 5 min, and then POD (conjugated with horseradish peroxidase) was dropped on the slides. The 3, 3'-diaminobenzidine (DAB) was used as the staining agent. All these process were performed according to the manufacturer's instructions (Roche Applied Science, USA).

### Superoxide measurement by DHE Staining

The cardiac tissues were embedded in optimal cutting temperature gel, sliced to 5 µm thick in a cryotome, and placed on glass slides. After application of 10 µM dihydroethidium (DHE,Life Technology, USA), tissue sections were incubated in a light-impermeable chamber at 37°C for 30 min. In the presence of superoxide, DHE is converted to the red fluorescent hydroxyethidium molecule. Images (×400 magnification) were captured.

### Measurement of NADP^+^/NADPH concentrations and ratio

NADPH oxidase activity was determined by the ratio of NADP^+^/NADPH. The concentration of NADP and NADPH were determined using EnzyChrom NADP^+^/NADPH assay kit (Bioassay Systems, CA). Manufacturer's protocols were followed. It based on a glucose dehydrogenase cycling reaction. The intensity of the reduced product color, measured at 565 nm, is proportionate to the NADP^+^/NADPH concentration in the sample.

### Estimation of superoxide dismutase (SOD), malondialdehyde (MDA) and glutathione peroxidase (GSH-Px) in heart tissue

The samples of heart tissue were weighed and homogenized (1∶10, w/v) in 50 mmol·L^−1^ phosphate buffer (pH 7.4). The SOD, GSH-Px activity and MDA level were measured by colorimetric analysis using a spectrophotometer with the associated detection kits (Nanking Jiancheng Bioengineering Research Institute, China).

### Immunohistochemical determination of Bcl-2, Bax and Caspase-3

Tissue sections were incubated with polyclonal primary antibodies (Santa Cruz Biotechnology, USA) of Bcl-2, Bax and Caspase-3 overnight at 4°C. After washing with PBS (phosphate buffer saline), the sections were incubated with biotinylated horse anti-mouse IgG for 30 min and then incubated with the avidin-biotin-peroxidase complex using an ABC kit. The reaction was visualized by color development with 3, 3'-diaminobenzidine tetrahydrochloride (DAB). After counterstaining with hematoxylin, the slides were dehydrated and mounted in permount. Images (×400 magnification) were captured. The staining was quantified with Image-Pro Plus v6 analysis software.

### Western blot analysis

Heart samples were homogenated by lysis buffer [20 mM Tris, pH 7.5, 150 mM NaCl, 1 mM EDTA, 1 mM EGTA, 1% Triton X-100, 2.5 mM sodium pyrophosphate, 1 mM β-glycerolphosphate, 1 mM Na_3_VO_4_, 1 µg/mL aprotinin leupeptin and pepstatin, 1 mM Phenylmethylsulfonyl fluoride (PMSF)] and then centrifuged at 12 000 rpm for 15 min at 4°C was collected, and the protein concentration was measured using the Bicinchoninic Acid (BCA) protein assay (Beyotime, China). Approximately 50 µg protein was loaded onto 10% or 12% SDS-PAGE and transferred to a nitrocellulose membrane. The membranes were blocked with 5% non-fat dry milk in Tris-buffered saline and incubated with different primary antibodies at 4°C overnight. The following antibodies were used in this study: Bcl-2, Bax, p67^phox^, P38 MAPK and phospho-P38 MAPK(Thr180/Tyr182), Jun NH2-terminal kinase (JNK) and phosphor-JNK (Thr183/Tyr185), ERK1/2MAPK and phosphor-ERK1/2 MAPK(Thr202/Tyr204) (Cell Signalling Technology, USA) used at a 1∶1,000 dilution and gp91^phox^, p47^phox^, p67^phox^ (Santa Cruz Biotechnology, CA) used at a 1∶200 dilution. The membrane was treated with horseradish peroxidase-conjugated secondary antibody for 1 h at 37°C. Blots were developed by ECL kit (Pierce Biosciences, USA). The intensity of protein band was semi-quantitative measured by image analysis software (GeneTools from SynGene).

### Statistical analysis

Data were presented as mean ± SD. Statistical analysis was performed using a one-way analysis of variance (ANOVA). Differences were considered to be significant at *P*<0.05.

## Results

### FPE ameliorated myocardial apoptosis in experimental diabetic mice

**Figure 1:**
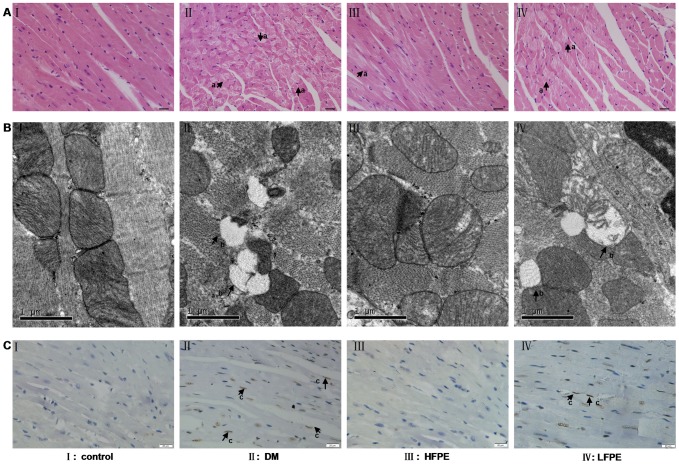
FPE ameliorated myocardial injury in experimental diabetic mice. A: Representative pictures of myocardial tissue sections stained with hematoxylin and eosin (magnification = 400×), n = 6 per group. Arrow ***a*** indicates perinuclear vacuolization, bar is 20 µm. B: Representative transmission electron micrographs of left ventricular specimens, n = 4 per group. Arrow ***b*** indicates swollen mitochondria. C: Representative pictures of the TUNEL assay, n = 4 per group, bar is 20 µm. HFPE: high dose, 200 mg/kg; LFPE: low dose, 100 mg/kg.

The microstructure of cardiac tissue was examined by H&E staining. The mice in DM group displayed obvious structural abnormalities including perinuclear vacuolization and disorganized swollen fibrils (Fig 1A). We further detected the ultrastructural change in the myocardium. As compared with well organized, typical symmetric myofibrils comprised of Z lines with diads, sarcomeres, elliptical nucleus, packed mitochondria beside the fibers in control group, mice in DM group showed degeneration, destruction and loss of myofibrils over sarcomere units, and cristae was lost in most of the mitochondria (Fig 1B). Additionally, apoptotic positive cells indicated by TUNEL staining were much higher in DM group compared with control group (Fig 1C). In contrast, as shown in Fig 1, those abnormalities mentioned above were greatly ameliorated by FPE treatment.

### FPE reduced blood glucose and normalized body weight in experimental diabetic mice

**Figure 2:**
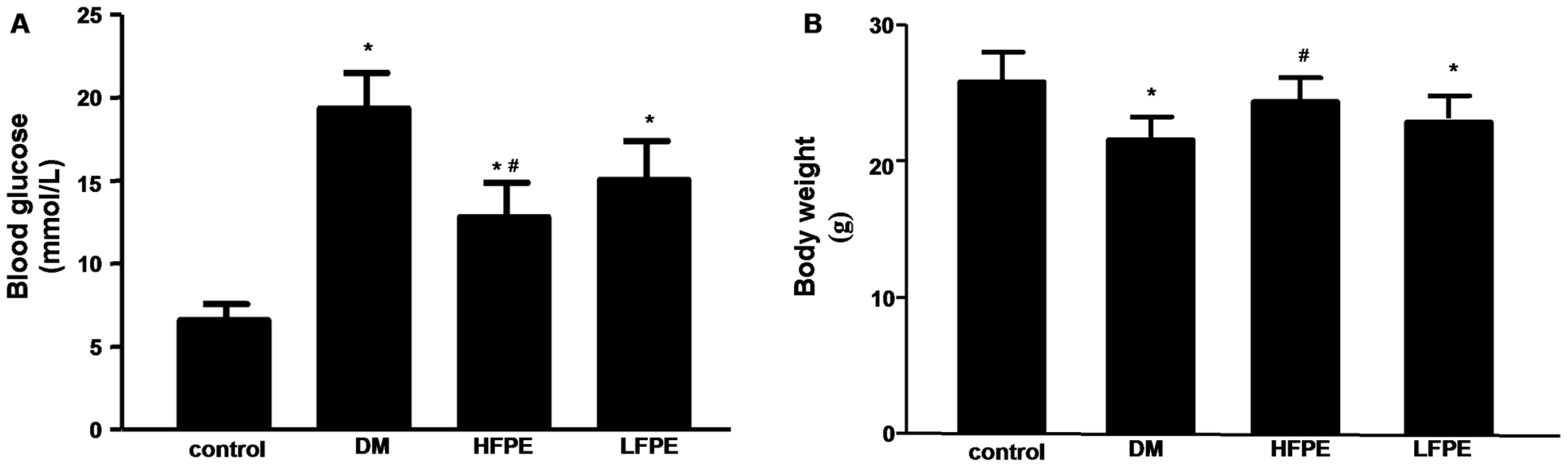
FPE decreased blood glucose and normalized body weight in experimental diabetic mice. A: FPE decreased blood glucose in the experimental diabetic mice. B: FPE increased body weights in the experimental diabetic mice. HFPE: high dose, 200 mg/kg; LFPE: low dose, 100 mg/kg. Blood glucose and body weight were measured in the basal fasting state on the day the mice were killed. Data are mean ± SD. ^*^ *P*<0.05 *vs* control group; ^#^ *P*<0.05 *vs* DM group, n = 10–12 per group.

The blood glucose significantly increased after injection with STZ in mice. And those with blood glucose>11.1 mmol/L were involved in the consecutive experiment. In parallel with the increased blood glucose, the experimental diabetic mice arose characteristic symptoms of diabetes such as polydipsia, polyuria, increased food intake and reduced body weight gain as compared with control mice (P<0.05). As shown in Fig 2, FPE supplementation markedly decreased the blood glucose level and normalized the body weight in experimental diabetic mice (P<0.05).

### FPE inhibited ROS production in cardiac tissue of experimental diabetic mice

**Figure 3:**
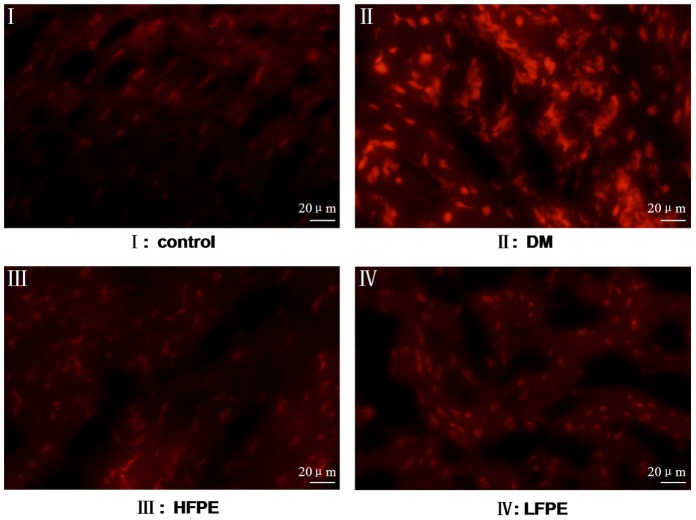
FPE decreased ROS production in experimental diabetic mice. Representative fluorescence images of heart sections stained with DHE(magnification = 400×), n = 3 per group, bar is 20 µm. HFPE: high dose, 200 mg/kg; LFPE: low dose, 100 mg/kg.

The superoxide generation in cardiac tissue was evaluated by DHE staining. As shown in Figure 3, superoxide production was significantly increased in the DM group compared to control group. And this elevation in ROS was markedly inhibited by FPE supplementation.

**Figure 4:**
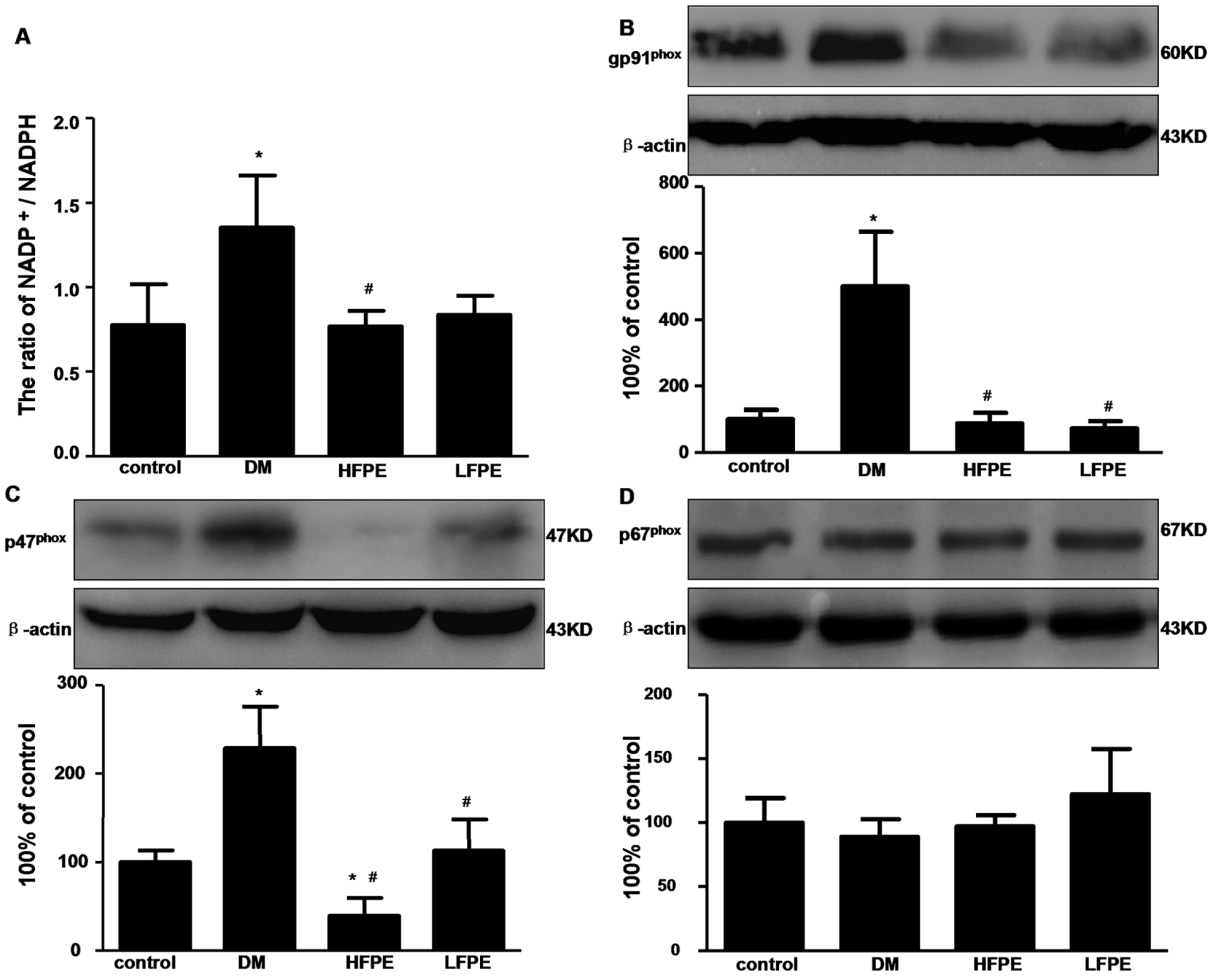
FPE inhibited NADPH oxidase activity and subunits expression in experimental diabetic mice. A: FPE decreased the ratio of NADP^+^/NADPH in experimental diabetic mice, n = 6 per group. B: western blots analysis of gp91^phox^, n = 3 per group. C: western blots analysis of p47^phox^, n = 3 per group. D: western blots analysis of p67^phox^, n = 3 per group. HFPE: high dose, 200 mg/kg; LFPE: low dose, 100 mg/kg. Data are mean ± SD. ^*^ *P*<0.05 *vs* control group; ^#^ *P*<0.05 *vs* DM group.

Next, we studied whether the NADPH oxidase is attributable to the increased ROS production. NADPH oxidase is an enzyme complex consisting of a lot of essential subunits such as gp91^phox^, p47^phox^ and p67^phox^. We measured the expression of these NADPH oxidase subunits in the heart of mice. As expected, cardiac expression of gp91^phox^ and p47^phox^ was increased in DM mice compared to the control mice, accompanied with the increased NADPH oxidase activity, as indicated by the increased ratio of NADP^+^/NADPH (P<0.05). In contrast, high dose FPE supplementation significantly reversed the alteration of NADPH oxidase activity and subunits expression (Figure 4, P<0.05). These results demonstrated that down regulation the NADPH oxidase subunits of gp91^phox^ and p47^phox^ by FPE treatment contribute to suppress ROS production in cardiac tissue of diabetic mice.

**Figure 5:**
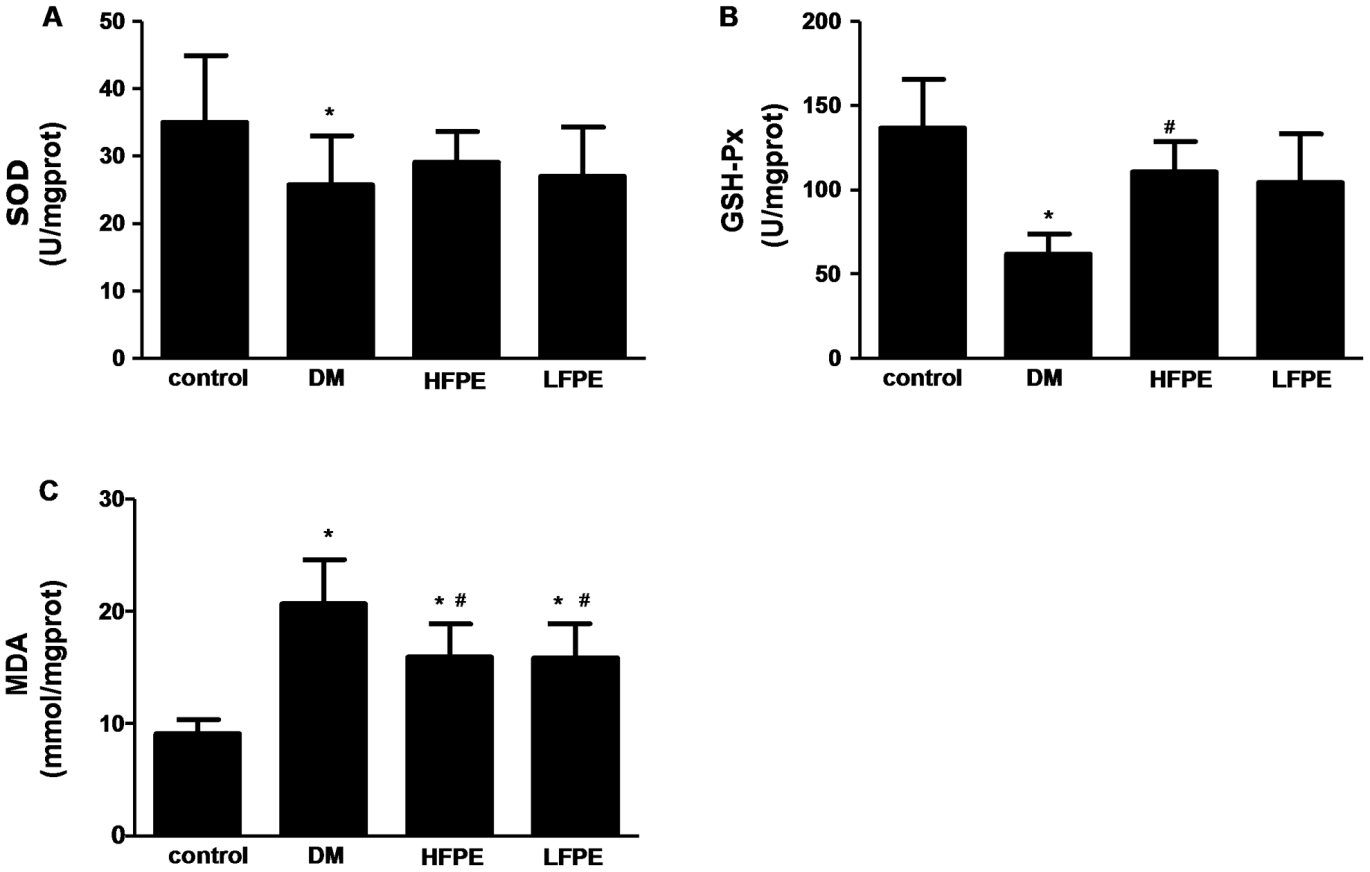
FPE reduced oxidative stress in experimental diabetic mice. A: FPE effected SOD activity in heart tissue. SOD: superoxide dismutase. B: FPE increased GSH-Px activity in heart tissue. GSH-Px: glutathione peroxidase. C: FPE decreased MDA content in heart tissue. MDA: malondialdehyde. HFPE: high dose, 200 mg/kg; LFPE: low dose, 100 mg/kg. Data are mean ± SD, ^*^ *P*<0.05 *vs* control group; ^#^ *P*<0.05 *vs* DM group, n = 10–12 per group.

Further, the cardiac activity of SOD and GSH-Px, two important antioxidant enzymes were markedly decreased in DM group compared to control mice (P<0.05).And the increased MDA level was also confirmed in DM mice (P<0.05). Those changes were attenuated by FPE supplementation in diabetic mice (Figure 5, P<0.05).

### FPE regulated the expression of Bcl-2, Bax and Caspase-3 proteins in experimental diabetic mice

**Figure 6:**
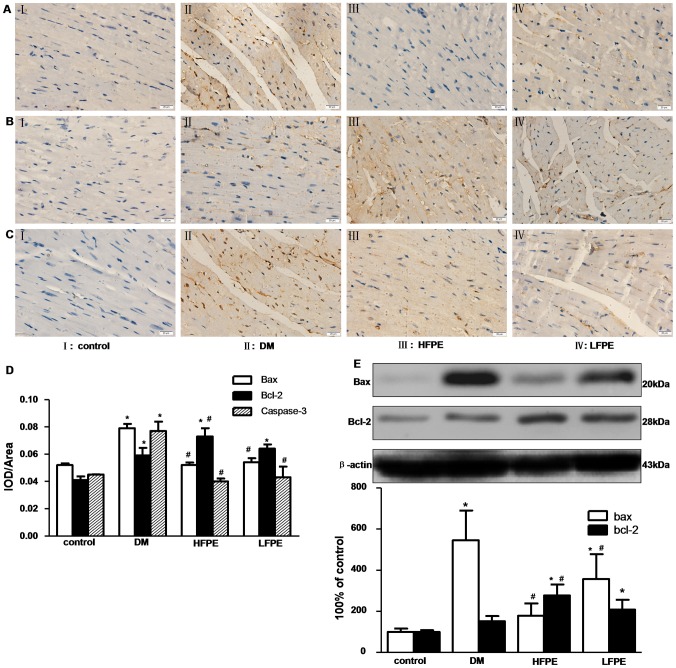
FPE regulated the expression of Bcl-2, Bax and Caspase-3 proteins in experimental diabetic mice. A–C: Representative immunohistochemical staining of Bax, Bcl-2 and Caspase-3 expression, respectively (magnification = 400×), n = 5 per group, bar is 20 µm. D: Representative immunohistochemical staining quantitative analysis of Bax, Bcl-2 and Caspase-3 expression. E: western blots analysis of Bcl-2 and Bax expression, n = 3 per group. HFPE: high dose, 200 mg/kg; LFPE: low dose, 100 mg/kg. Data are mean ± SD. ^*^ *P*<0.05 *vs* control group; ^#^ *P*<0.05 *vs* DM group.

It is well-known that apoptosis is regulated by series of apoptotic related proteins. Among them, Bax is accepted as a pro-apoptotic while Bcl-2 as an anti-apoptotic marker for apoptosis. And Caspase-3 is the executive marker for apoptosis. We further investigated whether FPE supplementation suppressed apoptosis through these regulatory proteins. As shown in Figure 6, Bcl-2 was down-regulated but Bax was up-regulated in the heart of DM group. And increased Caspase-3 expression was found in DM group. FPE treatment could notably down-regulate the ratio of Bax/Bcl-2 and Caspase-3 expression in the myocardium of experimental diabetic mice.

### FPE attenuates JNK and P38 MAPK activation in the heart of experimental diabetic mice

**Figure 7:**
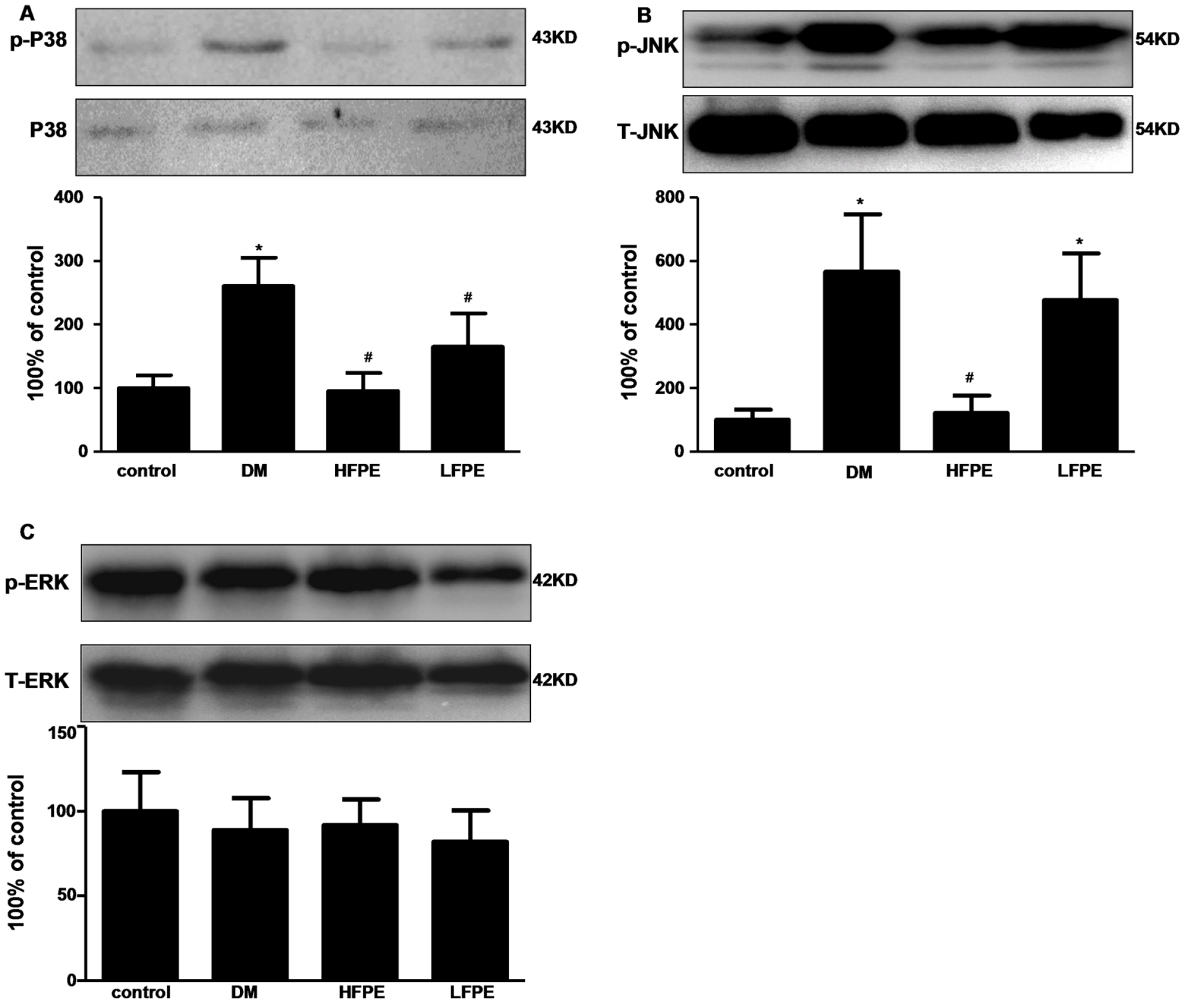
FPE attenuates mitogen activated protein kinases (MAPK) activation in experimental diabetic mice. A: western blots analysis of p-P38MAPK and P38MAPK. B: western blots analysis of p-JNK and JNK. C: western blots analysis of p-ERK1/2 MAPK and ERK1/2 MAPK. HFPE: high dose, 200 mg/kg; LFPE: low dose, 100 mg/kg. Data are mean ± SD, ^*^ *P*<0.05 *vs* control group; ^#^ *P*<0.05 *vs* DM group, n = 3 per group.

MAPK participates in a signaling cascade controlling cellular responses to stress. It is also reported to be powerful regulators of apoptosis [13].Three major subfamilies of MAPK signaling pathways included P38, JNK and ERK were evaluated in the heart of mice. As shown in Figure 7, P38 and JNK was markedly activated in the myocardial tissues of diabetic mice (P<0.05). FPE supplementation significantly abolished the activation of P38 and JNK (P<0.05). However, there was almost no detectable change for the phosphorylation of ERK in the myocardium among different groups.

## Discussion

Individual with diabetes remains at a significantly increased risk for heart failure even no concomitant risks such as coronary artery disease or hypertension [14]. This has led to the increased recognition of a unique clinical entity termed as “diabetic cardiomyopathy”, which was originally described by Rubler et al in 1972 [1]. In spite of increased structural heart disease due to both micro- and macro- vascular complications in diabetics, the concept of DCM defines a direct cellular insult to the myocardium secondary to diabetes [15]. Myocardial apoptosis is accepted as a key pathological change in DCM [16]. Previous studies have suggested that myocardial apoptosis increases and contributes to the loss of contractile units and reparative fibrosis in the heart of STZ-induced diabetic animals [17], [18]. Cardiac apoptosis is also believed to be a therapeutic target for DCM by various kinds of drugs [5], [19]. In the present study, we have shown myocardial apoptosis in the experimental diabetic mice, as indicated by destruction and loss of myofibril, swollen mitochondria and increased TUNEL positive cardiac cells. These structure abnormalities are consistent with previous reports [20]. And when the diabetic mice supplemented with the crude extract from *Flos Puerariae*, these cardiac structure injury and cell apoptosis mentioned above were greatly ameliorated.

Glucose has been considered as the main driving force for the development of DCM [21]. In the present study, FPE was administrated in the fourth week after STZ injection. We have demonstrated that high dose (200 mg/kg) of FPE significantly reduced the blood glucose level as well as weight profiles in the experimental diabetic mice, which is consistent with previous reports that total extract or isoflavonoid components of *Flos Puerariae* exert potent hypoglycemic effects in the STZ-induced diabetic rat [22], [23]. This hypoglycemic effect of FPE may retard the progression of DCM. However, recent clinical trials (UK Prospective Diabetes Study33 [UKPDS33], the Action to Control Cardiovascular Risk in Diabetes [ACCORD], the Action in Diabetes and Vascular Disease [ADVANCE] and the Veterans' Administration Diabetes [VADT]) [24] have revealed no significant effect of intensive glycemic control on mortality and amelioration of cardiovascular events [25]. Several other risk factors and mechanisms including oxidative stress also contribute to and exacerbate DCM. And the hypoglycemic effect of *Flos Puerariae* is also contributed mostly to its antioxidant action [22], [23].

As one of the most important factors involved in the pathogenesis of DCM, reactive oxygen species induced by hyperglycemia mediates its deleterious actions by affecting a series of downstream signal molecules that result in structural and functional cardiac changes [26]. Once the ROS production exceeds their scavenging by antioxidant defenses, oxidative stress occurs and the overwhelmed free radicals can damage DNA integrity, membrane lipids and protein function by oxidation and lead to functional abnormalities, apoptosis or necrosis [27]. It is well-known that one of the main sources of ROS production is the group of NADPH oxidase enzymes [28]. These enzymes act as catalysts for electron transfer from NADPH to molecular oxygen, resulting in generation of free radicals. Increasing evidence shows that NADPH oxidase subunits are transcriptionally upregulated leading to increased or prolonged ROS production in response to high glucose [29], [30]. Previous studies also demonstrated that NADPH oxidase-derived ROS generation is involved in the apoptosis of H9c2 cells and neonatal cardiomyocytes exposed to high glucose [31]. In the present study, increased superoxide formation has been detected in the cardiac tissue of diabetic mice. In parallel with that, NADPH oxidase subunits gp91^phox^ and p47^phox^ expression was significantly enhanced. On the contrary, the activity of antioxidant enzyme GSH-Px was reduced. And increased lipid peroxidation level has been conformed in the heart of diabetic mice. Our results further certified that *Flos Puerariae* extract could function as a suppressor of NADPH oxidase and ROS production in cardiac tissue of diabetic mice. And as the consequence of reduced ROS production by FPE treatment, the expression of apoptosis related proteins Bax/Bcl-2 and Caspase-3 were reversed in the hearts of diabetic mice.

MAPK signaling pathway, which includes three major subfamilies known as c-Jun N-terminal kinases (JNK), P38, and extracellular signal regulated protein kinases (ERK), have been reported to be important mediators for the cardiac remodeling process in diabetic cardiomyopathy and participate in the development of myocardial damage [32], [33]. Additionally, MAPK signaling pathways are also powerful regulators of apoptosis. Hyperglycemia-induced ROS may lead to MAPK activation [34]. Studies also have shown that JNK [4] and P38 MAPK [35] activation mediates apoptosis in human endothelial cells exposed to high concentrations of glucose. And pharmacological inhibition of P38 MAPK signaling can decrease the collagen content in DCM [36]. In the present study, there was a striking increase in the levels of active JNK (p-JNK) and P38 (p-P38) MAPK in the cardiac tissue of diabetic model mice, which is consistent with previous report that NADPH oxidase-derived ROS-induced apoptosis is mediated via increasing activity of JNK in cardiomyocytes exposed to high glucose [31]. However, the phosphorylation of ERK was little changed, which may be in that ERK is generally a survival and pro-growth factor. FPE treatment can significantly suppress the activation of P38 and JNK. Evidences have shown that pharmacological inhibition of P38 MAPK signaling by some antioxidants can prevent the development of DCM [37]. Thus, our results suggest that the protective effects of FPE on the myocardial injuries may be mediated by JNK and P38 MAPK signaling pathways.

A lot of naturally occurring flavonoids isolated from *Flos Puerariae* have been shown to exhibit antioxidant effects both in vitro and in vivo. Lee et al [23] and Han et al [38] reported that tectorigenin isolated from *Flos Puerariae* showed antioxidant effects on 1,1-diphenyl-2-pirylhydrazyl (DPPH) radical, superoxide anion radical, hydroxyl radical and lipid peroxidation in vitro by enzymatic and nonenzymatic methods. Lee et al also reported that water extracts from *Flos Puerariae* showed anti-oxidative effects through improving mRNA expression or activities of hepatic antioxidant enzymes in vivo [39]. In our present study the bioactive components contained in the crude extract from *Flos Puerariae* should include flavone glycosides, flavonoid C-glycosides, isoflavone glycoside, saponins, sterol glycoside, alkaloid, amino acids, sugars, and et al. Of those the content of total flavonoids has been confirmed more than 17.5% through our optimized extraction procure. And there are at least five primary isoflavones have been identified as irisolidone, genistein, daidzein, kakkalide and puerarin. We have demonstrated this crude extract from *Flos Pueraria* possess myocardial protective effect in DCM closely related to its antioxidant action. It is reasonable to speculate that this outcome is mainly attributed by the total flavonoids among the extract. However, the active constituents and more molecular mechanisms still need to be further defined in future.

In summary, we are the first to report that *Flos Pueraria* extract inhibits the expression and activity of NADPH oxidase, ROS production, prevent against oxidative stress induced myocardial apoptosis in the heart of STZ-induced diabetic animals, and this effect may be mediated by P38 MAPK/JNK pathway. Our results suggest that *Flos Pueraria* extract may have great therapeutic potential in the treatment of DCM.
